# Complementary and alternative medicine for the management of orthopaedic problems in Swiss Warmblood horses

**DOI:** 10.1002/vms3.64

**Published:** 2017-05-29

**Authors:** Catharina D. Lange, Shannon Axiak Flammer, Vinzenz Gerber, Ditte Kindt, Christoph Koch

**Affiliations:** ^1^ Institute of Equine Medicine Department of Clinical Veterinary Medicine Vetsuisse Faculty University of Bern Bern Switzerland

**Keywords:** back problems, complementary and alternative medicine (CAM), horse, lameness, orthopaedic problems

## Abstract

It appears that complementary and alternative medicine (CAM) is used increasingly often in horses for the assessment and treatment of suspected orthopaedic problems, especially back problems. The aim of this study was to determine the frequency of CAM use for the management of orthopaedic problems in a defined population of Swiss Warmblood horses. A total of 239 owners and caretakers of horses from a pre‐defined database were called by a veterinarian to participate in the survey. A standardized questionnaire was designed to determine, for each orthopaedic case, where the localization of the problem was (limb or back) and if conventional medicine or CAM was used for consultation and treatment. When CAM was employed, the CAM discipline and administrator (veterinarian or alternative therapist) was defined. A total of 222 cases in 170 horses with orthopaedic problems were identified. Sixty‐two horses were identified with a back problem, 96 horses with a lameness involving one or more limbs and 12 horses with a combined back problem and lameness. CAM was used commonly in this population (73.9%, 164 of 222) for both diagnostic workup and treatment of suspected orthopaedic problems, but was rarely administered by a veterinarian (12%, 27 of 222). In general, if a back problem was suspected by the owner, CAM was more frequently applied for diagnosis and treatment than in cases where a lameness was suspected; (91.9%, 68 of 74) vs. (64.9%, 96 of 148) (*P* < 0.001), respectively. Osteopathy was the most frequently applied CAM discipline. CAM was frequently used for diagnostic and therapeutic purposes in Swiss Warmblood horses with suspected orthopaedic problems. CAM practitioners were consulted predominantly if a back problem was suspected, and the majority of CAM practitioners were not veterinarians.

## Introduction

A healthy musculoskeletal apparatus is critical for success in equestrian sports. Sport horses both at international and amateur levels are known to be assessed and treated by Complementary and Alternative Medicine (CAM) practitioners in order to maintain a sound level of fitness or to address suspected problems resulting from athletic activity.

The American Veterinary Medical Association defines Complementary and Alternative Veterinary Medicine as a ‘heterogeneous group of preventive, diagnostic, and therapeutic philosophies and practices.’ Common therapies include aroma therapy, phytotherapy, magnetic field therapy, orthomolecular therapy, energy therapy, low‐energy photon therapy, acupuncture, homoeopathy, nutraceutical therapy, Bach flower remedy therapy and manual or manipulative therapy (https://www.avma.org/About/Governance/Documents/2014W_2013W_Resolution3_Attch2.pdf [accessed 28 Jan, 2016]). The increasing amount of available training programmes for equine practitioners in Europe for various CAM modalities (Delétraz *et al*. 2007) may be indicative of the demand for CAM in veterinary medicine. Studies have investigated the effectiveness and success of CAM treatment methods in horses (Faber *et al*. 2003; Gomez Alvarez *et al*. 2008; Sullivan *et al*. 2008) but to the authors’ knowledge, the extent of the demand for CAM investigations and treatments remains unknown.

The objective of this study was to assess the frequency of the use of CAM assessments and treatments for equine orthopaedic problems in a defined Swiss Warmblood population and to identify factors, which may influence the owners’ decision to utilize these methods. Additionally, an overview of who administers the CAM (veterinarian vs. non‐veterinarian) was desired. The authors hypothesized that horse owners would prefer conventional veterinary consultation and management for musculoskeletal conditions in the limbs, whereas CAM would be preferred over conventional medicine for treatment of suspected neck or back problems.

## Materials and methods

### Horses

The study group was originally defined for an investigation that used a standardized questionnaire designed to collect data regarding the potential hereditary components of recurrent airway obstruction in Swiss Warmblood horses (Gerber *et al*. 2009). The 761 horses for this original study were all Swiss Warmblood horses aged 5 years and older, bred and housed in Switzerland, and registered with the Swiss Equestrian Federation. Two influential stallions with confirmed history of recurrent airway obstruction, and a large number of their descendants and offspring, were chosen for investigation (Ramseyer *et al*. 2007). Of these 761 interviewed owners, 357 horses were selected because the owners stated an orthopaedic problem in the recurrent airway obstruction‐questionnaire.

### Questionnaire

Based on previously collected data (Ramseyer *et al*. 2007; Gerber *et al*. 2009), a standardized questionnaire (Tables 1 and Table S1) was developed. All telephone interviews were conducted by the same veterinarian (D.K.). Prior to contacting the owners, the interviewer was trained for consistency and the questionnaire was tested in German and French by performing practice interviews under the supervision of one of the co‐authors (V.G.) with three other co‐workers who had extensive experience in questionnaire‐based research from a previous study involving more than 400 horses (Ramseyer *et al*. 2007).

**Table 1:** Owner survey, translated into English, used to interview owners regarding the use of Complementary and Alternative medicine (CAM) to address orthopaedic problems in their Swiss Warmblood horses

Lameness					
Appearance and progression of lameness					
How often has your horse been lame or had a back problem?	□ Once	□ Several times			
Same reason?	□ Yes	□ No			
When did the lameness/problem appear?					
How long has your horse been lame?					
Where did the lameness appear?	□ Left Front	□ Right Front	□ Left Hind	□ Right Hind	□ Ataxia
	□ All 4 legs	□ Back	□ Neck	□ Sacroiliac joint	
What was the reason?	□ Trauma	□ Shoeing	□ Others		
Do you know which structure was injured?	□ Superficial Flexor Tendon	□ Suspensory ligament	□ Check ligament	□ Joint	
	□ Muscle	□ Others			
Would you describe the lameness as severe or mild?	□ Severe	□ Moderate	□ Mild	□ very mild	
Consultation/Diagnosis/Treatment					
Who was your first choice for consultation?	□ Veterinarian	□ Others			
Has your horse been examined by a veterinarian?	□ Yes	□ No			
Has your horse been examined by a non‐veterinarian?	□ Yes	□ No			
Has your horse been treated with CAM?	□ Yes	□ No			
Which diagnostic tools have been used by the veterinarian?					
Which diagnostic imaging tools have been used by the veterinarian?	□ Yes Ultrasound	□ Yes Radiography			
	□ Yes Scintigraphy	□ Yes CT	□ Yes MRI		
Has diagnostic local anaesthesia been used?	□ Yes	□ No			
Has the horse been treated with medication?	□ Yes	□ No			
If yes, how was the medication administered?	□ Local	□ Systemic			
Which additional CAM treatment has your horse been administered?					
Which therapies helped your horse?					
Is your horse still lame?	□ Yes	□ No			
Would you allow your horse to be examined at the Equine Clinic of the Vetsuisse Faculty of Berne, Switzerland to take part in a case study for a doctoral thesis?	□ Yes	□ No			

In the context of this study, a categorization was made to differentiate ‘lameness’ and ‘back problem’. The term ‘lameness’ referred to any gait abnormality caused by pathologies localized in one or more extremity, whereas ‘back problems’ included pathologies located at the cervical, thoracolumbar and sacroiliac regions. The questionnaire had two different sections: one for lameness and one for back problems which were utilized depending on whether the owners’ declared the horse has a lameness problem, a back problem, or both. The questionnaire was designed to obtain the owners’ interpretations of the clinical signs and the frequency and localization of the musculoskeletal problems. Further questions aimed to distinguish who was consulted for the clinical evaluation and therapy, which diagnostic tools were used and which types of therapies were implemented.

### Statistical analysis

Statistical analyses were performed using a dedicated program.^1^ A two‐sided *P* value < 0.05 was considered statistically significant. Continuous data are expressed as mean ± standard deviation. Chi‐Square tests and analyses of variance were used to compare proportions and continuous variables for differences in gender, colour, age and language and for, different methods of CAM, as appropriate. For multivariate analysis of the use of CAM and CAM as the first choice in consultation, logistic regression analyses were performed. From the full logistic regression model, a backward variable selection was applied based on Akaike's information criterion (AIC).

Although CAM includes a wide spectrum of therapies, only the disciplines that were given as an answer to the questions were included in the analysis. Although by definition physical therapy is a health profession, it was treated in this study as a discipline of CAM because it represents an alternative treatment to conventional medicine.

## Results

### Horses

**Figure 1:**
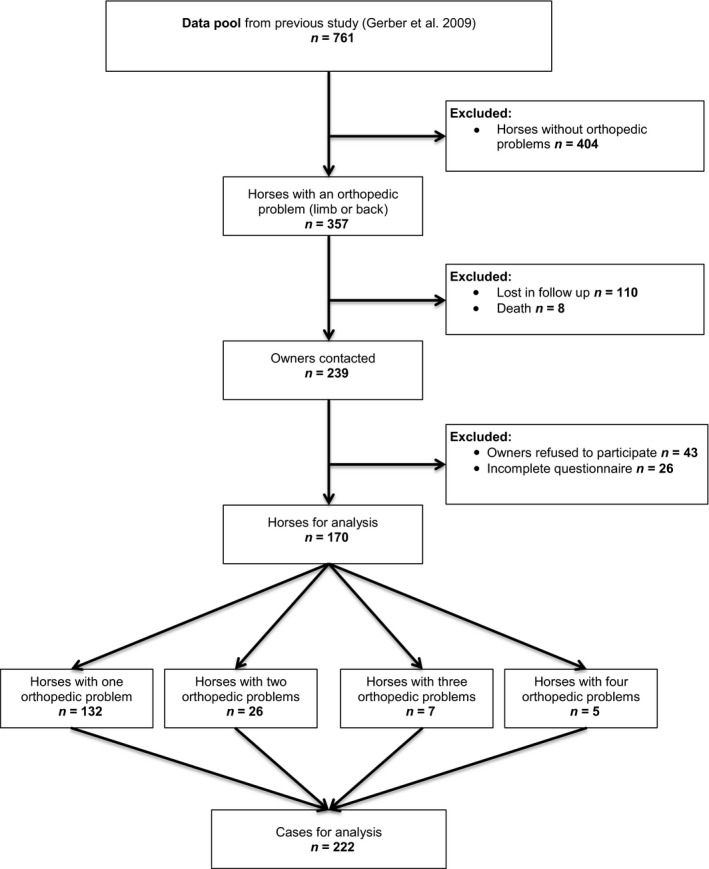
Case flow diagram (extra file)

Of the originally selected 357 horses, there were 170 eligible for analysis (Fig. 1). Of these, 62 (36.4%) horses had back problems, 96 (56.5%) horses had lameness on one or more limbs and 12 (7%) had both, back problems and lameness. In 132 (77.6%) horses, a single orthopaedic problem was identified, in 26 (15%) horses two orthopaedic problems, in 6 (3.5%) horses three orthopaedic problems and in 5 (2.9%) horses four orthopaedic problems. A total of 222 cases with orthopaedic problems were identified because each reported orthopaedic problem was considered as a separate case. The subsequent analyses are based on these 222 cases (Fig. 1).

Considering that approximately 80 000 Swiss Warmblood horses are registered in Switzerland (Poncet *et al*. 2009), the results drawn from the analyses of our study population compare to the actual population with an error level of 7.5% and a 95% confidence interval (CostumInsight Survey Random Sample Calculator).^2^ The demographic data of the horses are described in Table 2.

**Table 2:** Subject details for each orthopaedic group in an owner survey of the use of Complementary and Alternative medicine to address orthopaedic problems in Swiss Warmblood horses

	Total	Back	Lameness	Back and Lameness	*P* value
Cases (*n*)	170	62	96	12	
Gender (*n*)					
Stallion	1 (0.6%)	0 (0.0%)	1 (1.0%)	0 (0.0%)	0.9^b^
Gelding	92 (54.1%)	34 (54.8%)	52 (54.2%)	6 (50.0%)	
Mare	77 (45.3%)	28 (45.2%)	43 (44.8%)	6 (50.0%)	
Colour (*n*)					
Bay	101 (59.4%)	36 (58.1%)	59 (61.5%)	6 (50.0%)	0.3^b^
Chestnut	51 (30.0%)	16 (25.8%)	31 (32.3%)	4 (33.3%)	
Grey	13 (7.6%)	8 (12.9%)	3 (3.1%)	2 (16.7%)	
Paint	5 (2.9%)	2 (3.2%)	3 (3.1%)	0 (0.0%)	
Age (years)^a^					
Mean	14 ± 3	14 ± 4	14 ± 2	13 ± 2	0.6^c^
Owner language (*n*)					
German	127 (74.7%)	49 (79.0%)	69 (71.9%)	9 (75.0%)	0.6^b^
French	43 (25.3%)	13 (21.0%)	27 (28.1%)	3 (25.0%)	

a Age at first presentation (mean ± standard deviation).

b Chi‐square statistics.

c Analysis of variance.

### General use of CAM

In the 222 cases, CAM was used in 164 of them (73.9%). The dissemination of CAM was significantly more widespread for cases with suspected back problems (68 of 74, 91.9%) than for cases with lameness (96 of 148, 64.9%) (*P* < 0.001). Additionally, CAM was more often used in the French (47 of 55, 85.5%) speaking part of Switzerland than in the German (117 of 167, 70.1%) speaking regions (*P* = 0.02). There was a strong trend for older horses (>17 years) to receive CAM treatment compared to young horses. This finding was confirmed in multivariable logistic regression (Table 3).

**Table 3:** Multivariable analysis for the use of Complementary and Alternative Methods (CAM) in Swiss Warmblood horses

	Unadjusted^a^		Logistic regression, full model^b^		Logistic regression, variable selection^c^	
	OR (95% CI)	*P* value^d^	OR (95% CI)	*P* value^d^	OR (95% CI)	*P* value^d^
General use of CAM:						
Equine Gender						
Gelding or Stallion	Reference	0.04	Reference	0.03	–	
Mare	1.88 (1.02–3.55)		2.09 (1.07–4.21)		–	
Age (years)						
6–12	Reference	0.01	Reference	0.05	Reference	0.04
13–14	0.37 (0.14–0.86)		0.50 (0.18–1.27)		0.50 (0.18–1.27)	
15–16	0.29 (0.11–0.69)		0.30 (0.10–0.78)		0.30 (0.10–0.78)	
17–23	1.11 (0.29–5.46)		1.09 (0.25–5.73)		1.09 (0.25–5.73)	
Owner language						
German	Reference	0.02	Reference	0.005	Reference	0.005
French	2.58 (1.19–6.25)		3.27 (1.40–8.46)		3.27 (1.40–8.46)	
Orthopaedic problem						
Lameness	Reference	<0.001	Reference	<0.001	Reference	<0.001
Back	6.32 (2.76–17.18)		5.93 (2.47–16.68)		5.93 (2.47–16.68)	
CAM as first choice for consultation:						
Equine gender						
Gelding or stallion	Reference	0.8	Reference	0.6	‐	‐
Mare	0.93 (0.52–1.65)		0.84 (0.40–1.74)		‐	‐
Age (years)						
6–12	Reference	<0.001	Reference	0.02	Reference	0.02
13–14	0.30 (0.13–0.63)		0.54 (0.21–1.35)		0.53 (0.21–1.31)	
15–16	0.30 (0.13–0.67)		0.45 (0.17–1.16)		0.44 (0.17–1.12)	
17–23	1.99 (0.75–5.50)		2.72 (0.81–9.66)		2.67 (0.80–9.39)	
Owner language						
German	Reference	0.1	Reference	0.01	Reference	0.01
French	1.63 (0.85–3.07)		2.96 (1.28–7.10)		2.87 (1.25–6.83)	
Orthopaedic problem						
Lameness	Reference	<0.001	Reference	<0.001	Reference	<0.001
Back	13.44 (6.90–27.32)		13.63 (6.56–30.16)		13.47 (6.50–29.68)	

Odds ratios (OR) with 95% confidence intervals (Wald type).

a Univariate logistic regression analysis.

b Logistic regression analysis full model.

c Backward variable selection from full model Logistic regression.

d Likelihood ratio tests.

### Frequency of different CAM disciplines

Osteopathy was the most commonly used CAM discipline followed by homoeopathy and acupuncture. The distribution of the different CAM disciplines can be seen in Table 4. All disciplines of CAM were utilized more in horses with back problems.

**Table 4:** Frequency of applied complementary and alternative method (CAM) disciplines in a population of Swiss Warmblood horses

Method	Total	Back problem	Lameness	*P* value^a^
Cases (*n*)	222	74	148	
Osteopathy (*n*)	117 (52.9%)	47 (63.5%)	71 (47.7%)	0.03
Acupuncture (*n*)	33 (14.7%)	17 (23.0%)	16 (10.6%)	0.02
Homoeopathy (*n*)	50 (22.2%)	20 (27.0%)	29 (19.9%)	0.2
Physiotherapy (*n*)	25 (11.1%)	11 (14.9%)	14 (9.3%)	0.2
Chiropractic (*n*)	26 (11.6%)	13 (17.6%)	13 (8.6%)	0.05
Massage (*n*)	18 (8.0%)	6 (8.1%)	12 (7.9%)	1.0
Magnetic field Therapy (*n*)	12 (5.3%)	7 (9.5%)	5 (3.3%)	0.05
Animal communication (*n*)	3 (1.3%)	1 (1.4%)	2 (1.3%)	1.0
Kinesiology (*n*)	2 (0.9%)	1 (1.4%)	1 (0.7%)	0.6
Natural healer (*n*)	5 (2.2%)	3 (4.1%)	2 (1.3%)	0.2

a Chi‐square statistics.

### First consultation

CAM was the first choice in 66 cases (29.6%). Consultation of a CAM practitioner was the first choice for significantly more owners in cases with a suspected back problem (48 of 74, 64.9%) compared to cases with a lameness problem (18 of 149, 12.1%) (*P* < 0.001). This finding was confirmed in multivariable logistic regression (Table 3).

### Veterinary diagnosis

The owners declared that a veterinary diagnosis had been established in 75.7% (168 of 222) of all cases. When evaluating only lameness cases, owners reported that in 87.2% (130 of 149) of the cases, the diagnosis had been made by a veterinarian, whereas in only 51.4% (38 of 74) of cases with back problems (*P* < 0.001), a veterinarian clearly stated a diagnosis. A clinical examination had been conducted in 52.3% (116 of 222) of the cases and was supplemented by radiography in 24.8% (55 of 222), ultrasonography in 21.6% (48 of 222), scintigraphy in 2.7% (6 of 222) and CT as well as MRI in one case each. In the remaining 22.4% of the cases, the diagnoses were exclusively based on the owner's description of the clinical manifestations and/or reached by relying on imaging modalities alone. In horses with a lameness problem, radiography (44 of 151, 29.1% vs. 11 of 74, 14.9%, *P* = 0.02) and ultrasonography (46 of 151, 30.5% vs. 2 of 74, 2.7%, *P* < 0.001) were used more often compared to horses with a suspected back problem.

### Veterinary use of CAM

In total, 119 of the 222 cases were attended by both a veterinarian and a CAM practitioner. In only 27 of 222 cases (12%) was an alternative method administered by a veterinarian. These included 8 osteopathic treatments (3.6%), 8 chiropractic treatments (3.6%), 5 physiotherapy treatments (2.2%) and 4 magnetic field treatments (1.8%).

## Discussion

Complementary and alternative medicine was used to treat orthopaedic problems in nearly 75% of the examined cases. These survey results are comparable with results of a previous survey on the use of CAM in horses competing in equestrian disciplines in New Zealand (Meredith *et al*. 2011). In that survey, 62% of the 110 participants confirmed that their horses were treated with CAM to address a suspected back problem in 32% and lameness in 25% of the cases. In human medicine, Barnes *et al*. (2008) found that 40% of surveyed adults in the United States underwent some form of CAM therapy within a period of 12 months.

In this study, osteopathy was the most frequently applied CAM discipline followed by homoeopathy, acupuncture and others. In a survey conducted in European veterinary medical schools, Delétraz and colleagues reported acupuncture as the most commonly used CAM discipline in horses followed by homoeopathy, chiropractic and osteopathy (Delétraz *et al*. 2007). In a study from New Zealand, chiropractic was most frequently used in competition horses, followed by physiotherapy (Meredith *et al*. 2011). In these studies, the preference for CAM disciplines varied; therefore, we hypothesize that the choice for one specific alternative method depended on availability in the area of the owner or personal preference.

The majority of the owners initially consulted a veterinarian. If the problem did not resolve, they chose to consult a CAM practitioner. A CAM practitioner was the first choice for initial consultation in only one‐third of the cases; this tendency was more pronounced in horses suffering from suspected back problems. Jeffcott reported that thoracolumbar related pathologies and pain often manifest as reduced performance or rideability, which is difficult to assess clinically (Jeffcott 1975). Furthermore, back pain can often alter the gait and therefore can present as uni or‐ bilateral hindlimb lameness if the pathology is located in the thoracolumbar or sacroiliac region (Ranner & Gerhards 2001; Landman *et al*. 2004) or as front limb lameness if the pathology is located in the cervical region (Ricardi & Dyson 1993). Diagnosing the cause of a back problem in the horse is complex and is probably the reason why veterinarians were only able to reach a diagnosis in half of the back cases in contrast to horses with lameness where conventional diagnostic imaging was more applicable. Radiography and scintigraphy were used in horses with back problems, but in most cases the diagnosis was based solely on findings of the classical clinical examination as described by Jeffcott (1975).

Despite the predominance of CAM in our population, only a small percentage of CAM treatments were performed by a veterinarian. In Switzerland, there are no legal requirements for complementary medicine practitioners in animals and it is the respective private training institutions themselves that impose the requirements for certification. A survey regarding CAM dissemination in European Equine medicine (Delétraz *et al*. 2007) determined that 80% of all private, mostly international institutes for CAM do not require a veterinary degree to be eligible for training. Furthermore, only half of the surveyed universities offered complementary methods for students in any form and only four universities said they had research activity in this area. In a worldwide survey of CAM (Memon & Sprunger 2011), 16 of 34 veterinary faculties provided courses on CAM, but only one as a compulsory subject. Therefore, one of the reasons why veterinarians did not practice CAM frequently could be the lack of knowledge and training of CAM they received in veterinary school.

Our survey may be limited by the pre‐selection of our population. Furthermore, a survey is prone to subjectivity and misunderstanding. During the questioning, we realized that the difference between chiropractic and osteopathic treatment was not always clear to some owners. In the French speaking regions of Switzerland, manual therapy seemed to be equivalent to osteopathy, in the German speaking part, chiropractic. A reason for this may be that the most established training institutions for chiropractic (International Academy of Chiropractic (I.A.V.C.), [Link]) are located in Germany and the most established osteopathy schools are located in France (Formation vétérinaire en acupuncture et ostéopathie (AVETAO), [Link]). Therefore, the number of chiropractic and osteopathic cases may not be entirely accurate.

The results of this survey reflected the large demand for CAM by horse owners. The likelihood of CAM being used increased if a horse had back problems. A trained veterinarian administered CAM treatments in only a small number of cases. This underlines the need of an educational system, for people with non‐veterinary professional backgrounds, that assures adequate qualifications regarding equine anatomy and pathology, experience in handling horses, in addition to a thorough education in the CAM methods they are utilizing. It also shows the need for joint efforts to improve the synergistic use of conventional medicine and CAM and to develop new multidisciplinary approaches to equine orthopaedic problems. As veterinarians, we need to increase our understanding of the potential merits and limits of each CAM discipline and be able to critically assess their effects.

## Source of funding

This work was funded by the Swiss Institute of Equine Medicine (ISME).

## Conflict of interest

The authors declared that they have no conflict of interest.

## Ethics statement

The authors confirm that the ethical policies of the journal, as noted on the journal's author guidelines page, have been adhered to and the appropriate ethical review committee approval has been received.

## Contributions

None.

## Supporting information


**Table S1:** Original telephone questionnaires in German and French (extra file).

## Notes

1 The R Project for Statistical Computing (R‐statistical software V user! 2014, University of California, CA, USA) www.r-project.org [accessed 29 Jan, 2015).

2 Custom Insight Survey Random Sample Calculator, http://www.custominsight.com/articles/random-sample-calculator.asp/ [accessed 12 Nov, 2014].
