# Supplementary material for: Complementary and alternative medicine for the management of orthopaedic problems in Swiss Warmblood horses
Source: Vet Med Sci. 2017 May 29;3(3):125–33. doi: 10.1002/vms3.64 (PMC5645843; doi:10.1002/vms3.64)
Supplement: Supplementary file 1 — Table S1: Original telephone questionnaires in German and French (extra file). [file VMS3-3-125-s001.docx]

**Table S1:** Original telephone questionnaires in German and French

| **Zusatzfragebogen Lahmheit** | | | | | | |
| --- | --- | --- | --- | --- | --- | --- |
| **Auftreten der Lahmheit/Verlauf** | | | | | | |
| Wie oft war ihr Pferd schon lahm oder hatte es ein Rückenproblem? | □ Einmal | □ Mehrmals |  | |  |  |
| Gleiche Ursache? | □ Ja | □ Nein |  | |  |  |
| Wann ist die Lahmheit /das Problem aufgetreten? |  |  |  | |  |  |
| Wie lange war Ihr Pferd lahm? |  |  |  | |  |  |
| Wo ist die Lahmheit aufgetreten? | □ Vorne links | □ Vorne rechts | □ Hinten links | | □ Hinten rechts | □ Ataxie |
|  | □ Alle 4 Beine | □ Rücken | □ Hals | | □ Kreuzdarmbeingelenk |  |
| Was war die Ursache? | □ Trauma | □ Hufbeschlag | □ Andere | |  |  |
| Wissen Sie, welche Struktur betroffen war? | □ Beugesehne | □ Fesselträger | □ Unterstützungsband | | □ Gelenk |  |
|  | □ Muskel | □ Andere |  | |  |  |
| Würden Sie die Lahmheit als stark oder schwach beschreiben? | □ Stark | □ Mittelgradig | □ Leichtgradig | | □ Schwach |  |
| **Untersuchung / Diagnose/ Behandlung** | | | | | | |
| Wen haben Sie zuerst zu Rate gezogen? | □ Tierarzt | □ Andere |  |  | |  |
| Ist Ihr Pferd von einem Tierarzt untersucht und behandelt worden? | □ Ja | □ Nein |  |  | |  |
| Ist Ihr Pferd von einem Nichttierarzt untersucht und behandelt worden? | □ Ja | □ Nein |  |  | |  |
| Wurde das Pferd mittels alternativen Methoden behandelt? | □ Ja | □ Nein |  |  | |  |
| Welche diagnostischen Hilfsmittel wurden durch den Tierarzt verwendet? |  |  |  |  | |  |
| Wurden bildgebende Verfahren durchgeführt? | □ Ja Ultraschall | □ Ja Röntgen | □ Beides | □ Nein | |  |
|  | □ Ja Szintigraphie | □ Ja CT | □ Ja MRI |  | |  |
| Wurden diagnostische Anästhesien durchgeführt? | □ Ja | □ Nein |  |  | |  |
| Ist das Pferd mit Medikamenten behandelt worden? | □ Ja | □ Nein |  |  | |  |
| Wann ja, wie wurden die Medikamente verabreicht? | □ Lokal | □ Systemisch |  |  | |  |
| Welche zusätzliche(n) komplementären Behandlung(en) hat Ihr Pferd erhalten (CAM)? |  |  |  |  | |  |
| Welche Behandlung hat Ihrem Pferd geholfen? |  |  |  |  | |  |
| Ist Ihr Pferd noch lahm? | □ Ja | □ Nein |  |  | |  |
| Würden Sie Ihr Pferd eventuell im Rahmen eines Dissertationsprojektes an der Pferdklinik Bern untersuchen lassen? | □ Ja | □ Nein |  |  | |  |

| **Questions supplémentaires boiteries** | | | | | |  |  |  |  |
| --- | --- | --- | --- | --- | --- | --- | --- | --- | --- |
| **Apparition et dévéloppment de la boiterie** | | | | | |  |  |  |  |
| Combien de fois votre cheval a-t-il boité ou a-t-il eu des problèmes de dos ? | □ 1 fois | □ Plusieurs fois |  |  |  |  |  |  |  |
| L’origine de la boiterie/problem était-elle toujours identique ? | □ Oui | □ Non |  |  |  |  |  |  |  |
| Quand le problem est-il apparu ? |  |  |  |  |  |  |  |  |  |
| Combien de temps l’épisode de boiterie/problem a-t-il duré ? |  |  |  |  |  |  |  |  |  |
| Quelle était la localisation de la boiterie ? | □ Antérieur droit | □ Antérieur gauche | □ Postérieur droit | □ Postérieur gauche | □ Ataxique |  |  |  |  |
|  | □ Les 4 membres | □ Dos | □ Encolure | □ Articulation lumbo-sacrale |  |  |  |  |  |
| Quelle était la cause ? | □ Traumatisme | □ Ferrage | □ Autre |  |  |  |  |  |  |
| Savez-vous quelle structure était touchée ? | □ Tendon fléchisseur | □ Suspenseur du boulet | □ Bride accessoire | □ Articulation |  |  |  |  |  |
|  | □ Muscle | □ Autre |  |  |  |  |  |  |  |
| Comment décririez-vous l’intensité de la boiterie ? | □ Forte | □ Modérée | □ Faible | □ Très Faible |  |  |  |  |  |
| **Examen/Diagnostic/ traitement** |  |  |  |  |  |  |  |  |  |
| A qui avez-vous demandé conseil en premier ? | □ Vétérinaire | □ Autre |  |  |  |  |  |  |  |
| Votre cheval a-t-il été examiné et traité par un vétérinaire ? | □ Oui | □ Non |  | | |  |  |  |  |
| Votre cheval a-t-il été examiné et traité par une personne n`exerçant pas la médecine vététinaire? | □ Oui | □ Non |  |  |  |  |  |  |  |
| Votre cheval a-t-il déjà été traité par des méthodes alternatives (CAM) ? | □ Oui | □ Non |  |  |  |  |  |  |  |
| Quelles étaient les mesures diagnostiques et le diagnostic  de vétérinaire? Quelles méthodes ont été utilisées ? |  |  |  |  |  |  |  |  |  |
| Est-ce que des examens d’imageries ont déjà été effectués ? | □ Oui, échographie | □ Oui, radiographies | □ non |  |  |  |  |  |  |
|  | □ Oui, Scintigraphie | □ Oui, Scanner | □ Oui, IRM |  |  |  |  |  |  |
| Des anesthésies locorégionales ont elle été utilisées | □ Oui | □ Non |  |  |  |  |  |  |  |
| Est-ce que le cheval a reçu un traitement médicamenteux ? | □ Oui | □ Non |  |  |  |  |  |  |  |
| Si oui, comment le médicament etait donné ? | □ Local | □ Systémique |  |  |  |  |  |  |  |
| Quels traitements complémentaires ont été mis en place ? (physiothérapie, thérapie physique, méthodes alternatives, programme d’activité physique) |  |  |  |  |  |  |  |  |  |
| Quel traitement a été efficace pour votre cheval ? |  |  |  |  |  |  |  |  |  |
| Est-ce que votre cheval boite toujours ? | □ Oui | □ Non |  |  |  |  |  |  |  |
| Seriez éventuellement d’accord de laisser examiner votre cheval à la Clinque ISME de Berne pour une étude de doctorat ? | □ Oui | □ Non |  |  |  |  |  |  |  |
